# Genome-Wide Study of Plant-Specific PLATZ Transcription Factors and Functional Analysis of *OsPLATZ1* in Regulating Caryopsis Development of Rice (*Oryza sativa* L.)

**DOI:** 10.3390/plants14020151

**Published:** 2025-01-07

**Authors:** Tao Yang, Xin-Tong Xu, Li-Jun Tang, Wen-Tao Wei, Yuan-Yuan Zhao, Jin-Xin Liu, Xue-Feng Yao, Heng Zhao, Chun-Ming Liu, Ai-Ning Bai

**Affiliations:** 1 Key Laboratory of Plant Molecular Physiology, Institute of Botany, Chinese Academy of Sciences, Beijing 100093, China; yangtao@ibcas.ac.cn (T.Y.); xintong@stu.pku.edu.cn (X.-T.X.); ljtang0913@163.com (L.-J.T.); weiwentao@stu.ynu.edu.cn (W.-T.W.); zhaoyuanyuan231@mails.ucas.ac.cn (Y.-Y.Z.); jxliu@ibcas.ac.cn (J.-X.L.); yxfun@163.com (X.-F.Y.); cmliu@ibcas.ac.cn (C.-M.L.); 2 College of Life Sciences, University of Chinese Academy of Sciences, Beijing 100049, China; 3 School of Advanced Agricultural Sciences, Peking University, Beijing 100871, China; 4 School of Agriculture, Yunnan University, Kunming 650504, China; 5 Institute of Crop Sciences, Chinese Academy of Agricultural Sciences, Beijing 100081, China; zhaoheng@caas.cn

**Keywords:** PLATZ, photosynthetic organisms, rice, expression pattern, caryopsis development

## Abstract

Plant A/T-rich sequence- and zinc-binding protein (PLATZ) is a type of plant-specific zinc-dependent DNA-binding protein that binds to A/T-rich DNA sequences. This family is essential for plant growth, development, and stress response. In this study, 15 *OsPLATZs* were identified in the rice genome with complete PLATZ-conserved domains by CD-search, similar to those found in angiosperms. Multi-species phylogenetic analysis showed that PLATZs were conserved in photosynthetic organisms, and an evolutionary branch unique to angiosperms was identified among members of the PLATZ family. Fifteen OsPLATZs were represented by five groups, each with distinct characteristics. An analysis of protein structures and sequence motifs showed that OsPLATZs were similar within groups, but varied between them. The expression profile and qRT-PCR results showed that *OsPLATZs* had distinct expression patterns in different tissues, with some responding to stress induction. Most of the OsPLATZs localized to the nuclei, and were predicted to bind to DNA sequences by AlphaFold3, suggesting that they likely function as conventional transcription factors. We also identified *OsPLATZ1*, a caryopsis-specific gene that regulates grain filling and caryopsis development in rice. This research lays the foundation for exploring the structural diversity, evolutionary traits, expression profile, and possible roles of PLATZ transcription factors in rice.

## 1. Introduction

Transcription regulation is central to gene expression, as it precisely controls RNA production through DNA-binding transcription factors (TFs). This allows cells or organisms to respond effectively to developmental and environmental changes [1]. Usually, TFs act as switches controlling gene expression by binding to specific *cis*-elements upstream of target genes, thereby modifying their transcription [2].

TFs, whether in plants or animals, regulate a wide range of biological processes, including growth and development, metabolic regulation, immune response, and stress response [3]. The early divergence of plants and animals in the eukaryotic lineage gave rise to distinct transcription factor families, shaped by their unique evolutionary paths and functional needs over time [4]. In plants, specific transcription factor families like APETALA2/ETHYLENE RESPONSIVE FACTOR (AP2/ERF) [5], No apical meristem (NAM), Arabidopsis transcription activation factor 1/2 (ATAF1/2), CUP-SHAPED COTYLEDON 2 (CUC) (NAC) [6], myeloblastosis (MYB) [7], and WRKYGQK (WRKY) [8] are intrinsically linked to plant-specific physiological processes that respond to different environmental pressures. For example, the AP2/ERF family plays a pivotal role in regulating stress tolerance and development in plants [9], while the WRKY family is extensively involved in disease resistance and secondary metabolism regulation [8].

The PLATZ is a type of plant-specific TF family. *PLATZ1*, which was cloned in Pea (*Pisum sativum*), is the first reported member of the PLATZ protein family [10]. PLATZ1 contains two highly conserved zinc-finger motifs: C-X2-H-X11-C-X2-C-X (4-5)-C-X2-C-X (3-7)-H-X2-H (CHC4H2) and C-X2-C-X (10-11)-C-X3-C, and it can nonspecifically bind to A/T-rich sequences, leading to transcriptional repression [10].

Recent research has found that PLATZ TFs are vital for controlling the development and growth of plants, and also in stress response [11,12]. *AtPLATZ1* and *AtPLATZ2* play active roles in regulating seed desiccation tolerance [13]. T-DNA insertion mutants of *AtPLATZ1* and *AtPLATZ2* exhibit reduced germination rates due to decreased desiccation tolerance [13]. *ORESARA15*, also known as *AtPLATZ3*, controls root apical meristem (RAM) size by mediating the antagonistic interaction between auxin and cytokinin signaling-related pathways [14]. Moreover, *ORESARA15* can stimulate leaf development through boosting cell proliferation rate and duration in the early stages and suppressing leaf senescence later by modulating the GROWTH-REGULATING FACTOR (GRF)/GRF-INTERACTING FACTOR regulatory pathway [15]. *AtPLATZ4* positively increases plant drought tolerance through inhibiting the expression of *PIP2;8* and genes involved in ABA signaling in Arabidopsis [16]. RITF1 (AtPLATZ7) plays a key role in regulating the development of the Arabidopsis RAM by mediating Root meristem Growth Factor 1 (RGF1) signaling. Manipulating RITF1 expression redistributes reactive oxygen species (ROS) across the root developmental zones, which, in turn, enhances the stability of PLETHORA2 (PLT2), a key regulator of root stem cells [17]. *VviPLATZ1* plays a critical role in controlling female flower morphology in grapes, with loss-of-function mutants exhibiting reflex stamens, a defining feature of female flowers in *Vitis vinifera* [18].

PLATZ TFs play essential roles in seed development, not only in *Ginkgo biloba* (gymnosperm) [11], but also in food crops. In maize, the *Floury3* (*Fl3*) encodes a PLATZ protein and specifically expresses in starchy endosperm. Fl3 interacts with key components of the RNA polymerase III complex, RPC53 and TFC1, participating in the transcriptional regulation of tRNA and 5S rRNA, thereby influencing endosperm development and grain filling [19]. Similarly, *OsFl3* (*OsPLATZ1*), which is orthologous to maize *Fl3*, is involved in rice endosperm development, affecting grain size and filling rate [20]. GL6 (OsPLATZ11) also participates in RNA polymerase III transcription machinery by interacting with RNA polymerase III subunit C53 and TFC1 to regulate the expression of genes involved in rice grain development [21]. In addition, GmPLATZ controls seed size and weight by activating cyclin genes and *GmGA20OX*, promoting cell proliferation in soybean [22].

The endosperm tissue in rice and other grasses serves as an energy storage facility, and its proper development is essential for the synthesis and storage of nutrients [23,24]. These processes are governed by specialized TFs, including the PLATZ TFs, which play pivotal regulatory roles in the seed development of maize, rice, and soybean as introduced above [19,21,22]. However, there is no comprehensive overview of PLATZ TFs in rice currently.

In this study, we performed a systematic analysis of OsPLATZ TFs in rice, focusing on their genome-wide identification, chromosomal organization, and evolutionary relationships. By examining conserved motifs, *cis*-regulatory elements, and three-dimensional structures, we revealed key structural and functional features of OsPLATZ proteins. Additionally, expression profiling provided insights into their potential biological roles, highlighting candidate genes for future functional studies and crop improvement. Our findings offer new perspectives on the evolutionary and functional diversification of OsPLATZs, with potential applications in rice genetic improvement.

## 2. Results

### 2.1. Identification and Phylogenetic Classification of PLATZs in Photosynthetic Organisms

**Figure 1:**
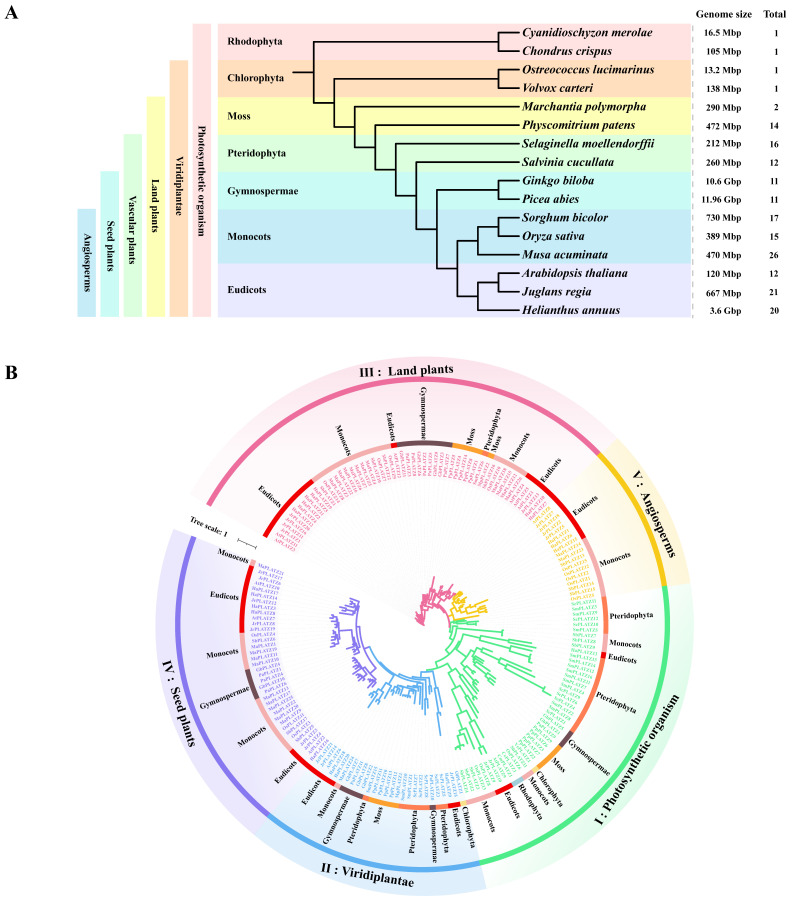
Comparison of PLATZ distribution and phylogeny among sixteen photosynthetic organisms across evolutionary time. (**A**) Sixteen selected photosynthetic species representing a broad range of evolutionary time. (**B**) Phylogenetic tree of PLATZ was constructed to display the evolutionary history of PLATZ TF families.

To better understand the evolutionary history of PLATZ proteins in plants, we selected 16 species from different lineages for analysis. These included two red algae, two green algae, two mosses, two ferns, two gymnosperms, and six angiosperms (three monocots and three eudicots). The genomes of all of these species have been sequenced. Among them, *Cyanidioschyzon merolae* (Cm) and *Chondrus crispus* (Cc) represent some of the earliest eukaryotic photosynthetic organisms, *Ostreococcus lucimarinus* (Ol) and *Volvox carteri* (Vc) are considered to be among the oldest unicellular green plants, *Marchantia polymorpha* (Mp) and *Physcomitrella patens* (Pp) represent early land plants, *Selaginella moellendorffii* (Sm) and *Salvinia cucullata* (Sc) represent ancient vascular plants, *Picea abies* (Pa) and *Ginkgo biloba* (Gb) represent the oldest gymnosperms. *Oryza sativa* (Os), *Sorghum bicolor* (Sb), and *Musa acuminata* (Ma) represent monocots, while *Arabidopsis thaliana* (At), *Helianthus annuus* (Ha), and *Juglans regia* (Jr) represent dicots (Figure 1A). In total, 181 non-redundant *PLATZ* genes were identified from these 16 species by searching for the PLATZ domain using the HMMER algorithm, and they were categorized into five major clades based on the maximum likelihood (ML) tree constructed by the FastTree tool (Figure 1B). Among these, the clade I PLATZs included those from photosynthetic organisms, implying that these orthologs are conserved among photosynthetic species and suggesting that the divergence of clade I PLATZs (PO group) occurred before diversification of these photosynthetic species. Clade II PLATZs (VP group) were found in all viridiplantae, from green algae to land plants examined in this study, indicating that the divergence of PLATZs in this clade may have occurred in the common ancestral species and play essential physiological roles for extant viridiplantae. Intriguingly, the PLATZ members significantly expanded in moss, which is consistent with clade III (LP group) having the maximum number of PLATZs, suggesting important functions for them in plant terrestrial adaptation. Within the clade III, a similar result could be observed, which also covered members from all land plants. Furthermore, clade IV PLATZs (SP group) were only present in the spermatophytes, implying that PLATZ orthologs in clade IV are conserved among seed plants and suggesting that their divergence occurred before the diversification of these species. Within clade IV PLATZs, *Picea abies* and *Ginkgo biloba* represented gymnosperms, which have a more primitive evolutionary relationship compared to angiosperms. Five PLATZ homologs from gymnosperms (PaPLATZ1, PaPLATZ4, PaPLATZ6, GbPLATZ4, and GbPLATZ10) were tightly clustered together with the clade IV PLATZs and placed in the outer layers of the clade, demonstrating that these primitive PLATZs may represent an ancient out-group relative to clade IV PLATZs in angiosperms. It is worth noting that clade V (AP group) only contains higher angiosperms rather than others and is a unique branch of angiosperms. The emergence of clade V may be crucial for the growth and development of angiosperms. In summary, these findings indicate that the PLATZ families are widely present in photosynthetic organisms and have undergone significant expansion in land plants. In addition, a branch that appears exclusively in angiosperms has differentiated.

Notably, the PLATZ TFs underwent rapid expansion with the emergence of mosses. Only one PLATZ member was found in each algal species from the most basal lineages, and two PLATZs were identified in liverwort *Marchantia polymorpha*. Starting from the moss *Physcomitrella patens*, members of this family began to expand significantly, with more than 10 PLATZs identified in ancient vascular plants, the oldest gymnosperms, and angiosperm (Figure S1). Additionally, the expression of some *PLATZs* acquired tissue specificity starting from moss *Physcomitrella patens* (Figure S2), indicating that the number of PLATZ family members increased significantly and some of them had specific expression patterns, which may be related to more complex organ differentiation in land plants.

### 2.2. Collinearity and Phylogenetic Analyses of PLATZs in Angiosperms

**Figure 2:**
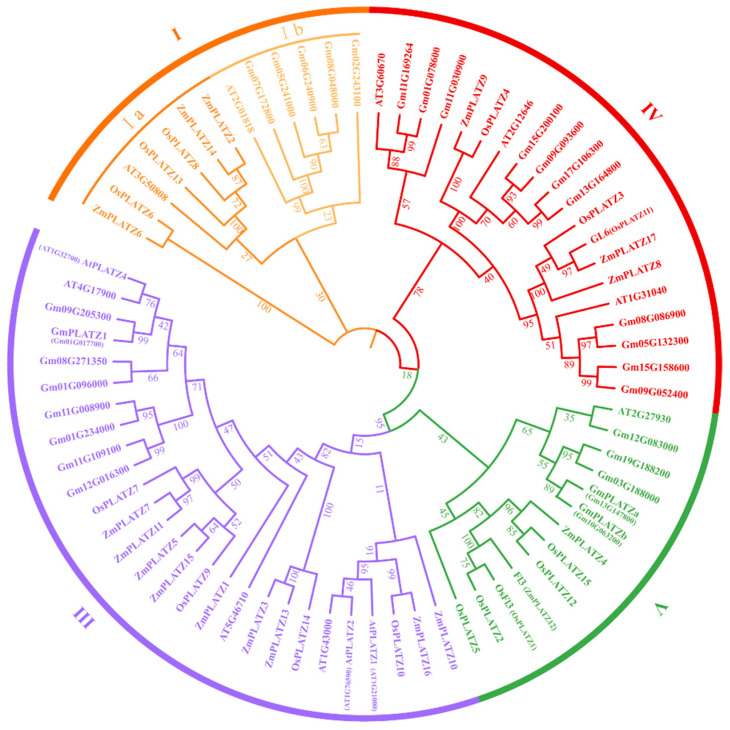
Phylogenetic tree of PLATZ proteins in *Oryza sativa*, *Zea mays*, *Arabidopsis thaliana*, and *Glycine max*. The prefixes Os, Zm, At, and Gm before the gene name represent *Oryza sativa*, *Zea mays*, *Arabidopsis thaliana*, and *Glycine max*, respectively. The numbers under the branches refer to the bootstrap values of the maximum likelihood phylogenetic tree. The four groups are represented by different colors.

To comprehend the evolutionary relationships of PLATZs in angiosperms, a phylogenetic tree was constructed using the maximum likelihood method and revealed distinct evolutionary relationships among 73 homologous amino acids from *Oryza sativa* (15 PLATZs), *Zea mays* (17 PLATZs), *Arabidopsis thaliana* (12 PLATZs), and *Glycine max* (29 PLATZs). All 73 PLATZ members were systematically grouped into four categories (I, III, IV, and V, without II) according to the topological arrangement of the phylogenetic tree and classification principles of photosynthetic organisms mentioned above in Figure 1B, with 13, 27, 20, and 13 members identified within clades I to V, respectively (Figure 2). Clade I was further divided into two sub-branches, Ia and Ib, corresponding to the photosynthetic organism clade and the green plant clade, respectively. Both mono- and dicotyledons existed in the Ia sub-branch, but only dicotyledonous plants were present in the Ib sub-branch. Interestingly, we did not find monocotyledonous plants from the *Poaceae* family in clade II, either in the evolutionary tree of photosynthetic organisms or in that of angiosperms. The members of clade II were all lost in rice, maize, and sorghum. This suggests a common evolutionary pressure or event that led to the loss of these members. Clade III had the most PLATZ members in all four species. Additionally, a closer evolutionary relationship was observed between *PLATZ* genes in rice and maize, as evidenced by their phylogenetic separation from soybean and Arabidopsis. Notably, some OsPLATZs were identified as orthologs of ZmPLATZs with a 100% bootstrap value. For instance, ZmPLATZ3 and ZmPLATZ13 were found to be orthologs to OsPLATZ14 (clade III), and ZmPLATZ9 was identified as an ortholog to OsPLATZ4 (clade IV), providing evidence for sequence conservation across species evolution. These findings shed light on the evolutionary dynamics and conservation patterns within the PLATZ family across different plant species.

**Figure 3:**
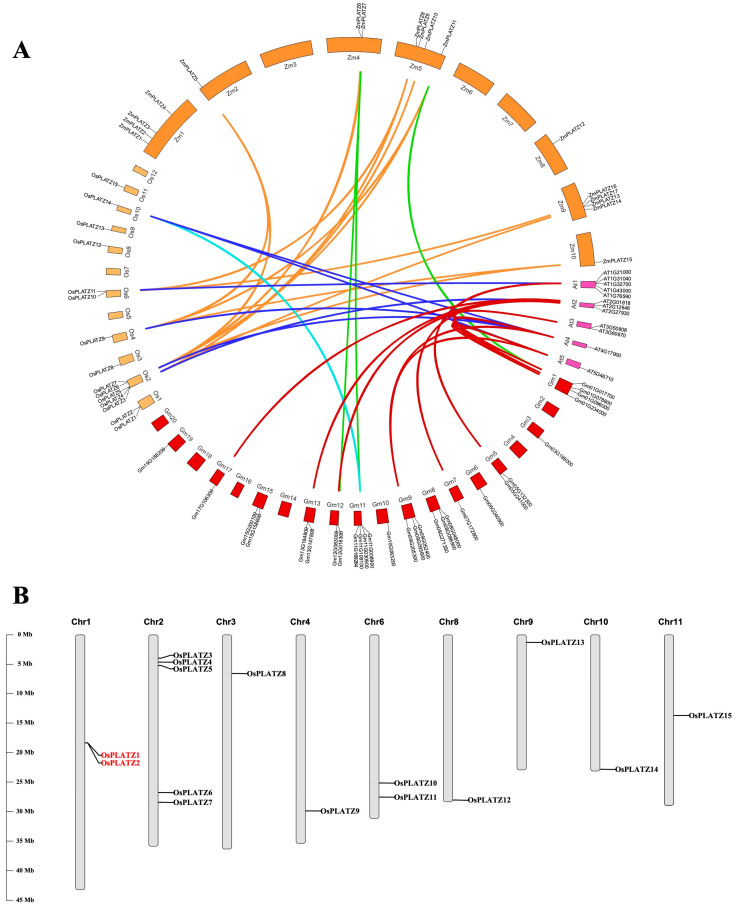
Collinearity analysis and mapping of *OsPLATZs*. (**A**) Collinear relationship analysis between orthologous *PLATZ* genes in rice, maize, Arabidopsis, and soybean. (**B**) Distribution and duplication of *OsPLATZ* genes in rice chromosomes. The tandem duplication event is marked in red. The chromosome numbers are shown at the top of each bar. The length of chromosomes was their relative extent. The scale on the left is in megabases (Mb).

The collinear relationships between 15 *OsPLATZs*, 17 *ZmPLATZs*, 12 *AtPLATZs*, and 29 *GmPLATZs* were analyzed for a better understanding of the duplication of *PLATZ* genes in monocotyledon and dicotyledon (Figure 3A). A total of seven *OsPLATZ* genes showed collinear relationships with those in maize, Arabidopsis, and soybean, indicating that these orthologous pairs may have already existed before the ancestral divergence. Overall, rice and maize show stronger collinearity, which was also observed between Arabidopsis and soybean.

The intraspecific gene duplication of *PLATZs* in soybean was more active than in the other three species (Figure S3), which explains very well why soybean contains far more PLATZ members than the other species. In rice, all 15 *OsPLATZs* are unevenly distributed across nine chromosomes, except for chromosomes 5, 7, and 12 (Figure 3B). *PLATZs* duplication occurred between chromosome 2 and other chromosomes (Figure S3A), explaining why one-third of the *OsPLATZs* are located on chromosome 2 (Figure 3B). Additionally, one pair tandem duplication (*OsPLATZ1* and *OsPLATZ2*) occurred on chromosomes 1 (Figure 3B). Apart from chromosome 1, 2, and 6, each contain more than two *OsPLATZs*, while chromosomes 3, 4, 8, 9, 10, and 11 each have only one *OsPLATZ* member (Figure 3B). The phenomenon of uneven distribution of *PLATZs* is also observed in maize, Arabidopsis, and soybean. The uneven distribution of *PLATZs* family members across chromosomes may result from multiple gene duplication events in different regions, reflecting an evolutionary strategy that allows species to adapt to diverse environmental pressures.

### 2.3. Conserved Motifs Among OsPLATZ Genes

**Figure 4:**
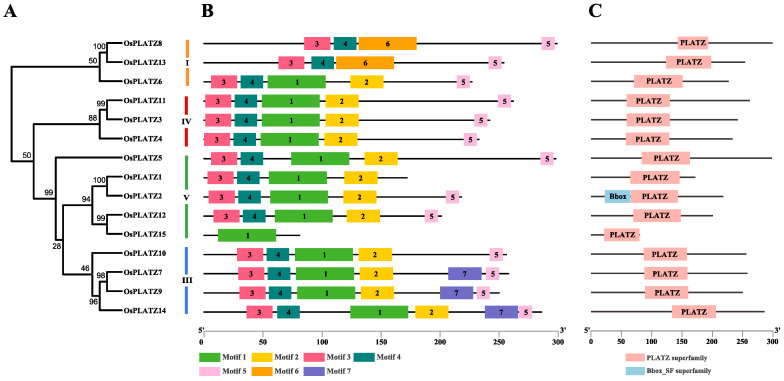
Phylogenetic relationship, motif structure, and conservation analysis of OsPLATZs. (**A**) Maximum likelihood phylogenetic tree of OsPLATZ proteins. I-IV: OsPLATZs were divided into four groups and are represented using different colors; (**B**) MEME motif structure shows the distinct divergence between groups; (**C**) Batch-smart analysis of PLATZ domain distribution of OsPLATZ proteins.

Due to the absence of the VP group, the phylogenetic tree of the OsPLATZs was divided into four clades as described above. The motif element analysis supports this classification (Figure 4A). Clades I and IV contained three OsPLATZs each, and four OsPLATZs were grouped into clade III. Clade V emerged as the largest, housing five OsPLATZs (Figure 4A). Seven motifs, designated motif-1 to motif-7, were identified through the MEME online tool (Table S1). The highly conserved distribution of the motifs within each group ensures accurate gene classification and the precise regulation of downstream genes. However, these motifs exhibited distinct divergence across four groups (Figure 4B). Motif-1, motif-2, and motif-6 overlap with the PLATZ domain (Table S1, Figure S4); furthermore, motif-3 and motif-4 overlap with the B-box domain, corresponding to the conserved cysteine and histidine residues in the N-terminal of PLATZ proteins (Table S1, Figure S4), which were found in all groups. Motif-5 corresponded to the termination region of PLATZs in all groups, suggesting the structural integrity of most PLATZs. The conserved motif-7 was only found in OsPLATZ7/9/14, which belong to clade III. All OsPLATZs contained PLATZ-conserved domains in the central region (Figure 4C and Figure S4), endowing them with the ability for zinc-dependent DNA binding.

### 2.4. Cis-Acting Element Analysis of OsPLATZs

**Figure 5:**
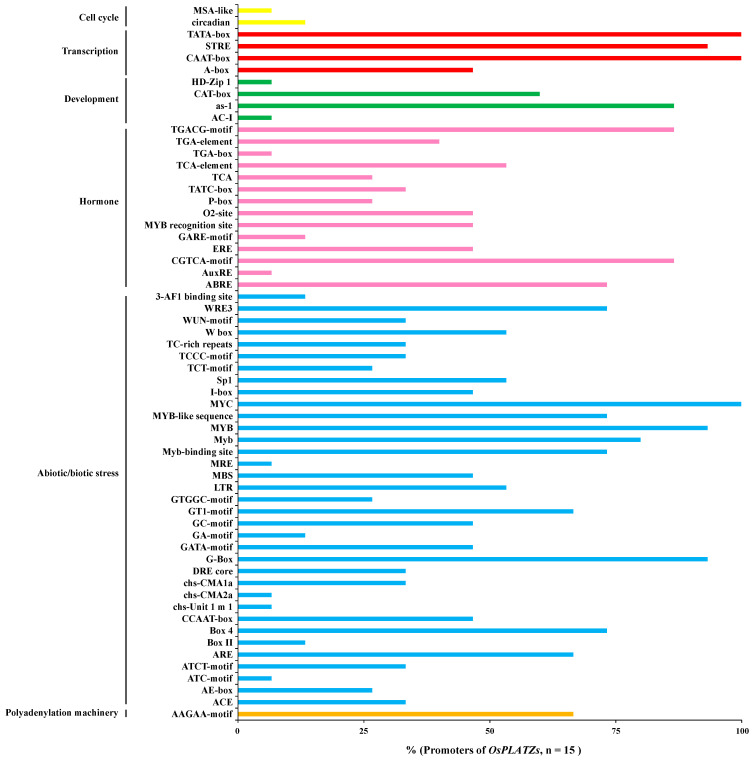
The ratio of *OsPLATZs* whose promoters contain certain types of *cis*-acting elements. *Cis*-acting elements were identified by PlantCARE using the 2 kb fragment from upstream of the transcription start site of each *OsPLATZ* gene. The graph was generated based on the ratio of *OsPLATZs* (*x*-axis) whose promoters contain certain types of *cis*-acting elements related to different conditions (*y*-axis). The *cis*-acting elements related to cell cycle, transcription, development, hormone, abiotic/biotic stress, and polyadenylation machinery are represented by different colors.

*Cis*-acting element analysis in the 5′ upstream regions of genes can provide useful clues for deciphering their function. Various *cis*-acting elements were identified in the 5′ upstream sequences of *OsPLATZs* predicted by PlantCARE, as shown in Figure 5 and Table S2. *Cis*-acting elements related to the cell cycle (MSA-like and circadian) were less prevalent, while four transcription-related elements (TATA box, CAAT-box, STRE, and A-box) were widely distributed in the 5′ upstream sequences of *OsPLATZs*. Four development-related elements, including AC-I, HD-Zip 1, as-1, and CAT-box, were also found in the 5′ *OsPLATZs’* upstream sequences, although not all *OsPLATZs* contained these elements, indicating functional differentiation in regulating their roles in plant development. Notably, most hormone related elements (ABRE, AuxRE, CGTCA-motif, ERE, GARE-motif, MYB recognition site, O2-site, P-box, TATC-box, TCA, TCA-element, TGA-box, TGA-element, and TGACG-motif) were retrieved in the 5′ *OsPLATZs* upstream sequences. Importantly, the elements related to biological and abiotic stresses were distributed abundantly in the 5′ upstream sequences of *OsPLATZs*, with 35 types observed, suggesting that *OsPLATZs* may play important roles in responding to different environmental signals. Moreover, AAGAA-motif, associated with the polyadenylation machinery, was found in two-thirds of the 5′ upstream sequences of *OsPLATZs*.

### 2.5. Protein Structures of OsPLATZs

**Figure 6:**
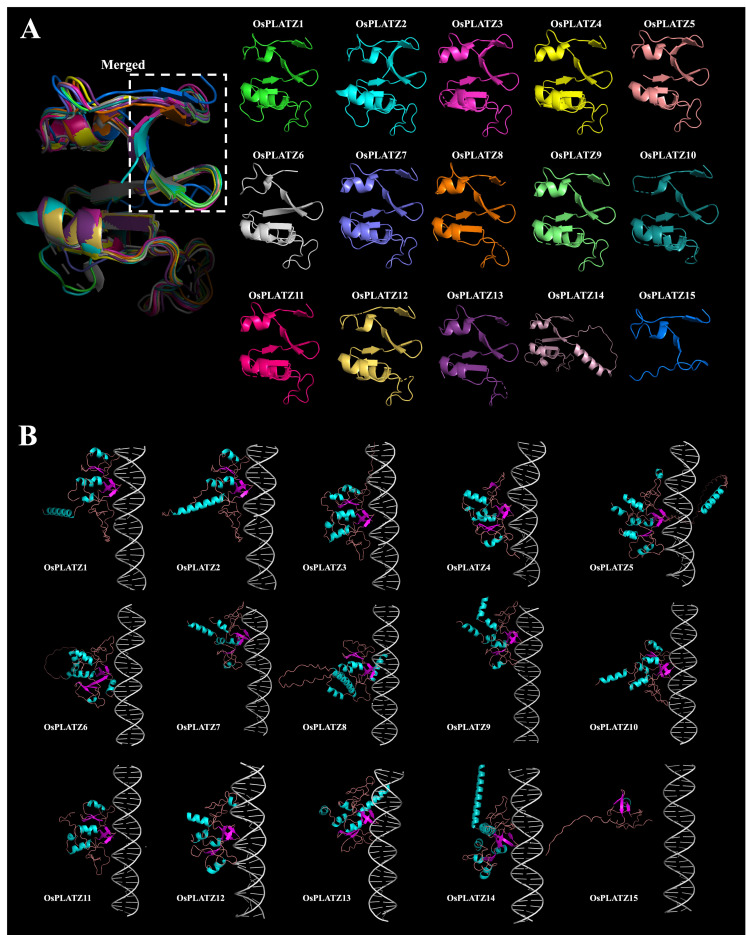
Prediction of the tertiary structures of OsPLATZ proteins and their binding to DNA fragments. (**A**) Protein 3D structure prediction of OsPLATZs. Different colors distinguish individual members, and regions with low confidence are hidden. The dashed box represents the merged diagram of the “C”-shaped structures of the 15 rice PLATZs. (**B**) Prediction of binding between OsPLATZs protein and DNA fragment, where light blue represents α-helices, magenta represents β-strands, and metallic colors represent random coils. DNA fragments with A/T rich sequences published in pea were used to carry out protein–DNA docking analyses. Protein tertiary structure prediction based on AlphaFold3.

Analyzing protein structures is crucial for understanding their functions. AlphaFold is a protein tertiary structure prediction algorithm with accuracy comparable to experimental methods such as CryoEM, NMR, or X-ray crystallography [11], and can rapidly predict the structures of multiple proteins. We used AlphaFold3 to predict the tertiary structures of all 15 rice PLATZs (Figure 6A) [25]. Tertiary structures, formed by the further coiling and folding of polypeptide chains from the secondary structure, are mainly stabilized by secondary interactions between amino acid side chains, including hydrophobic interactions, hydrogen bonds, Van der Waals forces, and electrostatic interactions. Through protein–DNA docking analysis, we found that all proteins except for OsPLATZ15 could successfully bind to DNA fragments with A/T rich sequences published in pea [10]. These proteins could fit into the DNA major groove through a conserved “C”-shaped structure (Figure 6B). This “C”-shaped structure consisted of four beta-sheet segments and two irregular coils. OsPLATZ15, due to the imperfect “C”-shaped structure lacking one beta-sheet, could not bind to the DNA fragment (Figure 6B).

### 2.6. Tissue Expression Patterns of OsPLATZs in Rice

**Figure 7:**
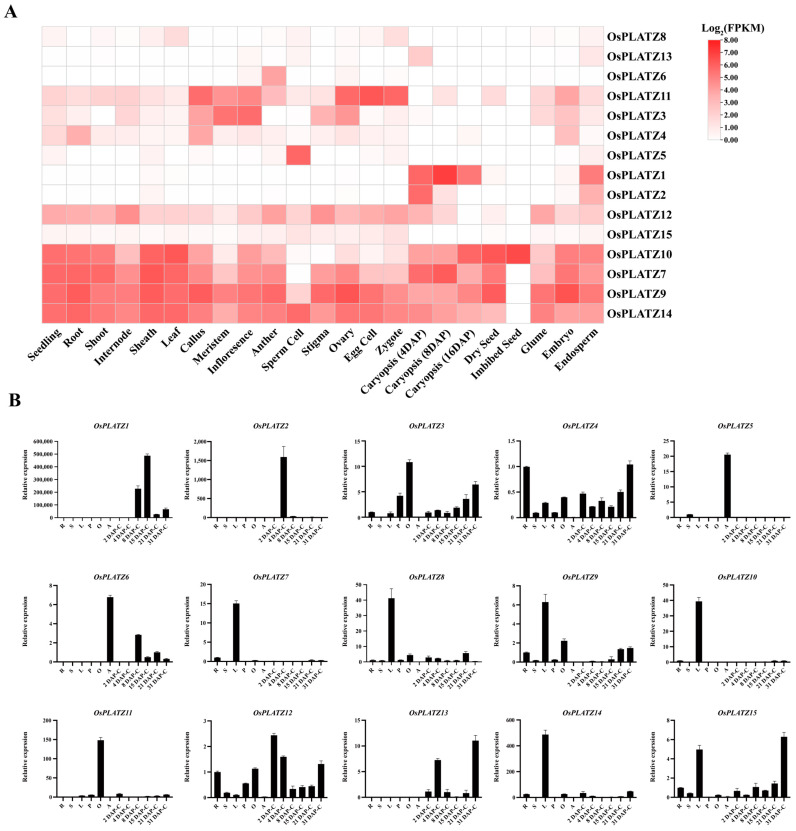
Expression patterns of *OsPLATZ* genes. (**A**) Expression patterns of *OsPLATZs* in different tissues analyzed using Plant Public RNA-seq data. The *OsPLATZ* genes are located on the right, and the tissues are at the bottom of each column. The color scale represents expression levels: red represents higher and white represents lower. The data values are shown as log_2_ (FPKM). (**B**) qRT-PCR of *OsPLATZs* in different tissues and stages. R, root; S, stem; L, leaf; P, 3-6 cm panicle; O, ovary; A, anther; C, caryopsis; DAP, day after pollination.

The spatiotemporal expression patterns of 15 *OsPLATZs* were characterized using transcriptome (Plant Public RNA-seq Database) data at different growth stages or in different tissues of rice [26]. In general, the expression patterns of *OsPLATZ* genes varied greatly in different tissues, indicating their potential involvement in multiple biological functions in rice (Figure 7A). Some *OsPLATZ* genes showed ubiquitous expression patterns, like *OsPLATZ7*, *OsPLATZ9*, *OsPLATZ10*, *OsPLATZ12*, and *OsPLATZ14*. In imbibed seeds, the expression level of *OsPLATZ10* was relatively higher. Both *OsPLATZ3* and *OsPLATZ11* were highly expressed in callus, meristem, and inflorescence, with *OsPLATZ11* also showing notable expression in the ovary, egg cell, and zygote. *OsPLATZ4* had an overall low expression level, with relatively higher expression in the root, callus, and embryo. The expression levels of *OsPLATZ8*, *OsPLATZ13*, and *OsPLATZ15* in all tissues were generally relatively low. Notably, some members showed tissue-specific expression patterns. *OsPLATZ5* was specifically expressed in sperm cells, and *OsPLATZ6* was specifically expressed in anthers. Both *OsPLATZ1* and *OsPLATZ2* were specifically expressed in the endosperm, exhibiting developmental specificity. *OsPLATZ1* began to express at 4 DAP, peaked at 8 DAP, and decreased after 16 DAP. Interestingly, *OsPLATZ2* was highly expressed at 4 DAP and then rapidly declined (Figure 7A). The relative quantification of *OsPLATZs* by qRT-PCR was similar to the RNA-seq expression profile (Figure 7B). However, due to sampling differences and batch effects in the public data, the quantitative results were inconsistent with the expression profile of transcriptome sequencing and require further analysis.

### 2.7. Subcellular Localization of OsPLATZs

**Figure 8:**
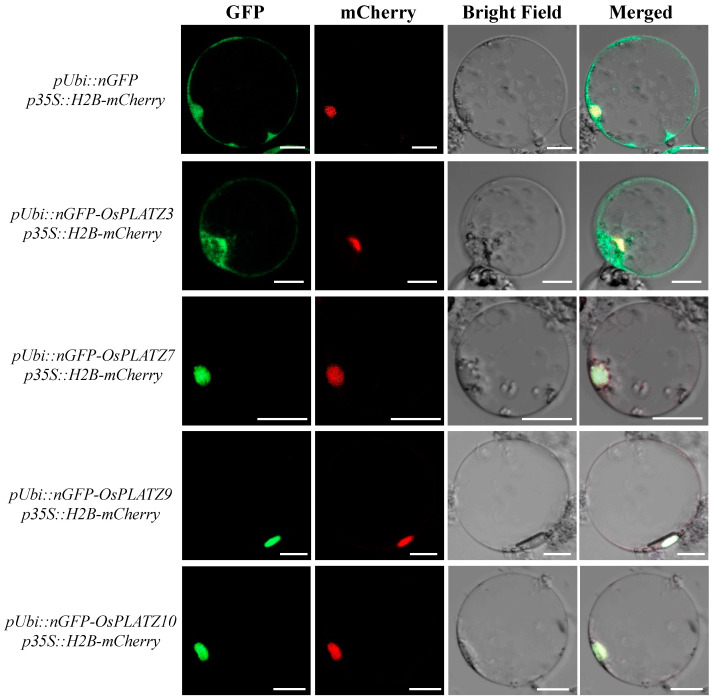
Subcellular localization of OsPLATZs in rice protoplasts. *pUbi::nGFP* as control, H2B-mCherry fusion protein as a nuclear localization marker. GFP, green fluorescence of fusion proteins; mCherry, monomeric cherry fluorescent protein; Merged, merged microscopic images. Scale bars = 10 μm.

Most PLATZ proteins in rice are predicted to be located in the nucleus (Table S3). To better understand the characteristics of these proteins, four OsPLATZs, OsPLATZ3/7/9/10, were randomly selected for subcellular localization analyses. As illustrated in Figure 8, the complete coding sequences of *OsPLATZ3/7/9/10* were cloned into the *pRTV-nGFP* vector under the control of the *Ubiqutin* promoter to construct vectors *pUbi::nGFP*-*OsPLATZ3/7/9/10*. Rice protoplasts were isolated and transiently co-transformed by *pUbi::nGFP-OsPLATZ3/7/9/10* plasmids with the *p35S::H2B-mCherry* plasmid, which expresses a nucleus-localized protein, respectively. The signal of the control vector *pUbi::nGFP* was detected in the nucleus and cell membrane, and consistent expression patterns were observed in OsPLATZ3 (Figure 8). The GFP signals of OsPLATZ7/9/10 were observed in the nuclei and colocalized with the red fluorescence H2B-mCherry fusion protein. These results suggested that OsPLATZ proteins are located in the nuclei to participate in various biological processes.

### 2.8. The OsPLATZ1 Mutant Showed Defective Endosperm

**Figure 9:**
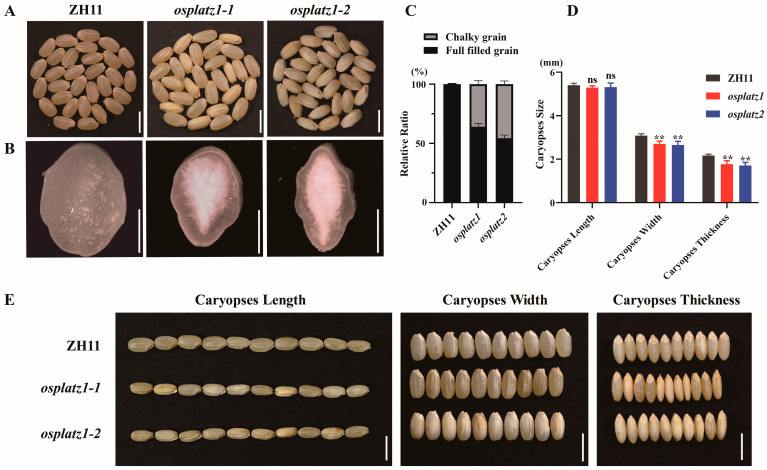
Phenotypic analyses of *OsPLATZ1* knockout mutants. (**A**) Mature caryopses of ZH11, *osplatz1-1*, and *osplatz1-2*. Bars = 5 mm. (**B**) Surface views of cracked mature caryopses from ZH11, *osplatz1-1*, and *osplatz1-2*. Bars = 1 mm. (**C**) Chalky caryopses percentage of ZH11, *osplatz1-1*, and *osplatz1-2* were quantified. *n =* 30, ** *p* < 0.01. (**D**) Statistical analysis of caryopses length, width, and thickness of ZH11, *osplatz1-1*, and *osplatz1-2*. *n =* 30, ** *p* < 0.01. ns, not significant. (**E**) Comparisons of 10-caryopses length, width, and thickness of ZH11, *osplatz1-1*, and *osplatz1-2*. Bars = 5 mm.

*OsPLATZ1* is specifically expressed in the endosperm (Figure 7A,B). To investigate the role of *OsPLATZ1* in endosperm development, we generated *OsPLATZ1* knockout lines in the ZH11 background using CRISPR/Cas9 technology. Two frameshift knockout lines, *osplatz1-1* and *osplatz1-2*, were successfully identified, with a single-base-pair insertion and a 12-base-pair insertion followed by 257-base-pair deletion, respectively (Figure S5). Phenotypic analysis showed that, compared to the semi-transparent caryopses in ZH11, most of the mature caryopses from *osplatz1-1* and *osplatz1-2* were chalkiness (Figure 9A). Additionally, the cracked caryopses of *osplatz1-1* and *osplatz1-2* displayed chalky endosperm in the central region (Figure 9B). Statistical analysis showed that the chalky caryopsis percentages of *osplatz1-1* and *osplatz1-2* (64.04% and 54.32%, respectively) were significantly lower than ZH11 (99.47%; Figure 9C). Furthermore, compared to ZH11, the caryopsis width and thickness, but not the length, of *osplatz1-1* and *osplatz1-2* were obviously decreased (Figure 9D,E). In summary, these findings highlight that *OsPLATZ1* is crucial for proper endosperm development and plays a significant role in regulating rice grain size and filling, as reported previously [20].

## 3. Discussion

PLATZs, a type of plant-specific zinc-dependent DNA-binding protein, are widely distributed among photosynthetic organisms. PLATZs play crucial roles in plant growth, development, and stress response [12,19]. However, the evolutionary dynamics and functional differentiation of PLATZs in rice have received relatively limited attention.

In this study, 181 PLATZs were identified from 16 species representing a broad range of evolutionary timescales. The copy number of PLATZ family proteins varies significantly among different plant lineages, ranging from a single copy in the most basal plant, *Cyanidioschyzon merolae*, to 26 copies in *Musa acuminata* (Figure 1). No direct correlation was found between the number of genes in a family and the sizes of their respective genomes. For instance, *Musa acuminata* has 26 PLATZs within a 470 Mbp genome, whereas *Picea abies* has 11 PLATZs with a genome size of 11.96 Gbp. In addition, no significant difference in the number of PLATZs was found between *Salvinia cucullata* and *Ginkgo biloba*, despite their clearly different genome sizes of 260 Mbp and 10.6 Gbp, respectively (Figure 1).

The identification of PLATZ members in 16 representative photosynthetic organisms revealed that PLATZs were present as a single member in both *Rhodophyta* and *Chlorophyta*, respectively, but underwent significant expansion from mosses to angiosperms. This expansion phenomenon is not unique to the PLATZ family; similar expansions have been observed in other gene families, including MCM1, AGAMOUS, DEFICIENS and SRF (MADS-box) [27], Late Embryogenesis Abundant (LEA) [28], Heat Shock Proteins (HSPs) [29], and NAC [6]. All of these families underwent significant amplification, starting from mosses and continuing in higher plants. Some members have acquired specific expression patterns and participate in particular biological processes or new organogenesis, adapting to the more complex challenges of terrestrial environments, such as drought, heat, and UV radiation [30,31]. For the PLATZ family, clade V (AP group) is a unique evolutionary branch within angiosperms, suggesting that it may be a newly evolved member that emerged during evolution (Figure 1B). More importantly, some members analyzed in rice, such as *OsPLATZ1*, *OsPLATZ2*, and *OsPLATZ5*, showed specific expression patterns in the anther or endosperm (Figure 7A,B), suggesting that *PLATZs* unique to angiosperms may be involved in regulating flower formation and seed development.

Gene duplication, including tandem duplication and segmental duplication, serves as the primary driving force for gene expansion [32,33]. In rice, we found one (*OsPLATZ1/2*) pair of tandem duplicated (Figure 3B) and three pairs of segmentally duplicated *OsPLATZs* (Figure S3A). These duplicated genes always show similar gene structures, but prefer to have different expression patterns and may acquire new functions during evolution. *OsPLATZ1* (*OsFl3*) and *OsPLATZ2* are homologous to *ZmPLATZ12* (*Fl3*) [19,20]. Although both were specifically expressed in the endosperm, they displayed differences in their temporal expression patterns. *OsPLATZ1* was specifically expressed in caryopses at the grain filling stage, while *OsPLATZ2* was specifically expressed in caryopses at the cellularization stage (Figure 7A,B). Additionally, the functional analysis showed that *OsPLATZ1* was involved in regulating grain filling. The mutants of *osplatz1-1* and *osplatz1-2* showed a chalky endosperm and significant reductions in caryopses width and thickness (Figure 9). However, whether *OsPLATZ2* redundantly regulates rice caryopses development or obtain differentiated functions from *OsPLATZ1* still needs to be elucidated in future studies.

Most of the OsPLATZs were predicted to be localized in the nuclei, and we randomly selected four members to confirm these results by the subcellular localization experiments (Figure 8). Additionally, previous studies have reported that PLATZs can bind to DNA fragments with A/T-rich sequences [10]. Here, we used AlphaFold3 to predict the tertiary structures of all 15 OsPLATZs (Figure 6A). Through protein–DNA docking, we found that the conserved “PLATZ” domain from 14 OsPLATZs (except for OsPLATZ15) could form a “C”-shaped structure composed of four beta-sheet segments and two irregular coils, which perfectly embed into the DNA major groove (Figure 6B). This suggests that OsPLATZs may play roles as transcription factors, binding to the promoter regions and regulating the expression of target genes. Notably, the “PLATZ” domain in OsPLATZ15 could not form a typical “C”-shaped structure and therefore could not bind to DNA fragments, implying that it may not function as a typical transcription factor.

## 4. Materials and Methods

### 4.1. Identification of Putative PLATZ Gene Families in Photosynthetic Organisms

The genome datasets of 16 representative photosynthetic organisms were retrieved from public databases with the primary sources being EnsemblPlants (http://plants.ensembl.org/index.html, accessed on 15 September 2024) [34], Phytozome version 13.0 (https://phytozome-next.jgi.doe.gov/, accessed on 15 September 2024) [35], TAIR (https://www.arabidopsis.org/, accessed on 15 September 2024) [36], the Rice Genome Annotation Project (https://rice.uga.edu/, accessed on 15 September 2024) [37], Ginkgo DB (https://ginkgo.zju.edu.cn/, accessed on 15 September 2024) [38], Evorepro (https://evorepro.sbs.ntu.edu.sg/species/, accessed on 15 September 2024) [39], and Fernbase (https://fernbase.org/, accessed on 15 September 2024) [40].

Pfam domain models of PLATZ proteins (PF04640) were downloaded from the Pfam database (http://pfam.xfam.org/, accessed on 16 September 2024) [41]. The predicted corresponding putative PLATZ homologs were retrieved using a HMMER search against the genome sequence in each plant with a threshold of E-value < 10^−5^. Some genes would contain multi-transcript isoforms because of alternative splicing. Thus, we removed all redundant candidates, and the remaining candidates were subsequently further validated separately using SMART (http://smart.embl.de/smart/batch.pl, accessed on 16 September 2024) [42], CDD (https://www.ncbi.nlm.nih.gov/Structure/bwrpsb/bwrpsb.cgi, accessed on 16 September 2024) [43], and Pfam (http://pfam.xfam.org/search, accessed on 16 September 2024) [41], in normal mode. Sequences with the PLATZ domain were ultimately retained for further analysis. The names of putative *PLATZ* genes were identified among these 16 organisms and assigned based on their corresponding chromosomal order (Table S4).

### 4.2. Multiple Sequence Alignments and Phylogenetic Analyses

Multiple sequence alignments of the above retrieved PLATZ candidate protein sequences were conducted using Clustalw2 version 2.1 in the Linux operating system with default parameters [44]. The results of multiple sequence alignments were displayed using ESPript 3.0 online tools (https://espript.ibcp.fr/ESPript/cgi-bin/ESPript.cgi, accessed on 17 September 2024) [45]. The ML tree was constructed using the FastTree tool (version 2.1) with the parameter “JTT + CAT” and 1000 bootstrap replicates [46]. The final ML tree was visualized using the iTOL version 6 online tool (https://itol.embl.de/, accessed on 17 September 2024) [47] and further polished with AI software (version 2023). In addition, a phylogenetic tree of 16 representative photosynthetic organisms was built based on TIMETREE (version 5.0) online servers (http://www.timetree.org/, accessed on 10 September 2024) [48].

### 4.3. Conserved Motifs, PLATZ Domains, and Putative Cis-Acting Elements of OsPLATZs

Conserved motifs of OsPLATZ proteins were identified using the MEME website (https://meme-suite.org/meme/tools/meme, accessed on 15 May 2024) [49] with the following parameters: distribution of motifs, 0 or 1 occurrence per sequence; maximum number of motifs, 7; minimum sites, 6; maximum width, 50.

Conserved domains of OsPLATZs were searched against the CDD version 3.21 database with the assistance of the NCBI CDD search tool (https://www.ncbi.nlm.nih.gov/Structure/cdd/wrpsb.cgi, accessed on 15 May 2024) with the following parameters: E-value threshold, 0.01; maximum number of hits, 500. TBtools-II software [50] was used to visualize the results of conserved motifs and conserved domains of 15 PLATZ members in rice.

The 2 kb fragment from upstream of the transcription start site of each *OsPLATZ* was extracted as a promoter to predict *cis*-acting elements using PlantCARE (http://bioinformatics.psb.ugent.be/webtools/plantcare/html/, accessed on 16 October 2024) [51]. Then, statistics derived from hits of various *cis*-acting elements were constructed and displayed in a diagram.

### 4.4. Location of OsPLATZ Genes on the Chromosome and Collinearity Analysis of OsPLATZs

The genomic locations of the 15 *PLATZ* members in rice were extracted from the rice genome annotation file (https://rice.uga.edu/pub/data/Eukaryotic_Projects/o_sativa/annotation_dbs/pseudomolecules/version_7.0/all.dir/all.gff3, accessed on 28 September 2024) and visualized using TBtools-II software (version 2.121) based on their chromosome locus and the length of each chromosome [50].

The duplication gene pairs in the PLATZ family were identified by Diamond BLASTP with an E-value of 10^−5^ and a maximum of five alignment targets [52]. MCScanX was then applied with default parameters to detect tandem [53] and segmental duplications of *PLATZ* genes among 4 species (Table S5), and the syntenic relationships were plotted using the Tbtools-II software (version 2.121) [50].

### 4.5. Protein Property Analysis

ExPASy (http://web.expasy.org/compute_pi/, accessed on 15 May 2024) [54] was used to predict the physical and chemical parameters of OsPLATZs (Table S3), including molecular weight (MW) and theoretical isoelectric point (PI). AlphaFold3 [25] was used to predict the tertiary structure of proteins, A/T rich sequence from pea [10] was used to predict binding with OsPLATZs and The PyMOL Molecular Graphics System (version 3.0 Schrödinger, LLC, New York, NY, USA) was used to visualize the results [55].

### 4.6. Expression Profiles of OsPLATZs

Public RNA-seq data were used to analyze the gene expression patterns of *OsPLATZs*. FPKMs of 15 *OsPLATZs* in different tissues [26] were collected and integrated via Excel software (version 2023), and a heatmap was drawn with the help of TBtools-II software (version 2.121) [50].

All 15 *OsPLATZ* genes were quantified by real-time quantification PCR (RT-qPCR) in roots (1-month-old), stems (1-month-old), leaves (1-month-old), panicle (3–6 cm), ovaries (before pollination), anthers (before pollination), and caryopses within different days (2, 4, 8, 15, 21, and 31) after pollination (DAP) from Zhonghua 11 (*Oryza sativa* L. ssp. *japonica* cultivar Zhonghua 11, ZH11), with three biological replicates. The total RNA from fresh tissues were extracted using TRIzol reagent (Invitrogen, San Diego, CA, USA) and then digested with RNase-free DNaseI. The first-strand cDNAs were synthesized using FastKing RT Kit (with gDNase; TIANGEN, KR116, Beijing, China) following the manual instructions. Gene-specific primers were designed using NCBI Primer-BLAST (https://www.ncbi.nlm.nih.gov/tools/primer-blast/index.cgi?LINK_LOC=BlastHome, accessed on 11 March 2024). The housekeeping gene *OsActin1* was used as an endogenous reference (all primers are shown in Table S6). The RT-qPCR was performed with the iTaq Universal SYBR^®^ Green Supermix (Bio-Rad, Hercules, CA, USA) in a CFX Connect real-time PCR detection system (Bio-Rad) according to the manufacturers’ instructions, with three technical replicates for each reaction. PCR amplification was performed under the following conditions: 95 °C for 30 s; 95 °C for 5 s, 60 °C for 30 s, and 72 °C for 15 s with 40 cycles; 95 °C for 10 s. Relative expression levels were calculated using 2^−ΔΔCt^. Data analysis and visualization were performed using GraphPad Prism version 8.0.0 for Windows (GraphPad, San Diego, CA, USA).

### 4.7. Prediction and Validation of OsPLATZs Subcellular Localization

Amino acid sequences were used to predict the subcellular localization of a total of 15 OsPLATZs using Plant-mPloc [56]. To verify the prediction results, four *OsPLATZs*, randomly selected, were tested by protoplast subcellular localization. The CDS of these genes (all primers are shown in Table S6) were constructed into the *pRTV-nGFP* vector [57], to obtain *pUbi::nGFP-OsPLATZ3/7/9/10* constructs. H2B-mCherry fusion protein was driven by the *35S* promoter as a nuclear localization marker and *pUbi::nGFP* as a control. The high-quality plasmids mentioned above were extracted using an EndoFree Maxi Plasmid Kit V2 (TIANGEN, DP120, Beijing, China) according to the manufacturer’s instructions. The rice protoplasts were isolated from albino seedlings cultured in a dark environment in an incubator for 12–14 days after germination. For transformation, *pUbi::nGFP-OsPLATZ3/7/9/10* plasmids were co-transformed transiently with *p35S::H2B-mCherry* mediated by PEG4000 solution for 30 min, and then the protoplasts were cultured at 25 °C in the dark for 14 h before observation. Each transient transformation experiment was repeated at least three times. GFP fluorescence was recorded using an ultra-high-resolution confocal microscope (Zeiss LSM 980 with Elyra7, Oberkochen, Germany).

### 4.8. Generations of Knockout Mutants Using CRISPR/Cas9

Clustered regularly interspaced short palindromic repeat (CRISPR)/CRISPR-associated nuclease 9 (CRISPR/Cas9) constructs were made according to Liu et al. [58], using the guide sequences for candidate genes designed by the web tool CRISPR-GE (http://cbi.hzau.edu.cn/cgi-bin/CRISPR, accessed on 10 November 2021) [59], and transformed into ZH11 via *Agrobacterium*-mediated transformation [60]. Sanger sequencing (Sangon Biotech, Beijing, China) was performed to identify homozygous mutants. PCR products amplified with HygR-F and HygR-R primers (primers are shown in Table S6) were used to select transgene-free lines.

## 5. Conclusions

In this study, a total of 15 *OsPLATZs* distributed across nine chromosomes were identified in the rice genome, which is similar in number to those in maize. OsPLATZs showed more collinearity with ZmPLATZs, but almost no collinearity with Arabidopsis or soybean, which is consistent with the relatively distant evolutional relationship between monocotyledons and dicotyledons. The phylogenetic analysis revealed that OsPLATZs can be divided into four groups, with rice, corn, and sorghum all losing clade II. The analysis of the conserved domains and sequence motifs highlighted distinct sequence characteristics for each group, with members of group III showing significant differences compared to the others. Based on the comparison with the *cis*-acting element database, *OsPLATZ* genes appear to be regulated by a variety of hormonal and environmental factors, and several response elements related to plant development and growth. The prediction of tertiary structures and protein–DNA binding showed that the conserved “PLATZ” domain from 14 OsPLATZs (excluding OsPLATZ15) forms a “C”-shaped structure, which aligns perfectly with DNA major groove. Importantly, we identified *OsPLATZ1*, a caryopsis specifically expressed gene, which is involved in regulating grain filling and caryopsis development in rice. This study sheds light on the conservation and evolutionary dynamics of PLATZ TFs in angiosperms, providing valuable insights into their regulatory roles in rice caryopsis development.

## Data Availability

The datasets presented in this study can be found in online repositories. The names of the repository/repositories and accession number(s) can be found in the article/Supplementary Material.

## Supplementary Materials

The following supporting information can be downloaded at https://www.mdpi.com/article/10.3390/plants14020151/s1: Figure S1: The PLATZ types were classified according to the phylogenetic analysis. Figure S2: The expression pattern of *MpPLATZs* (A) and *PpPLATZs* (B) from public data. Figure S3: Intra-genomic collinear relationship analysis of orthologous *PLATZ* genes in rice, maize, Arabidopsis, and soybean. Figure S4: Amino acid sequence alignment of 15 OsPLATZs. Figure S5: The information of *osplatz1* mutants created by CRISPR/Cas9. Table S1: The amino acid sequences and logo motifs for the seven characterized motifs. Table S2: The number of *cis*-acting elements in the 15 *OsPLATZs* promoters. Table S3: Analysis of physical and chemical properties of OsPLATZs. Table S4: Putative *PLATZ* genes identified in 16 organisms. Table S5: PLATZ proteins from Arabidopsis, soybean, and maize used in this study. Table S6: Primers used in this study.
