# Supplementary material for: Genome-Wide Study of Plant-Specific PLATZ Transcription Factors and Functional Analysis of OsPLATZ1 in Regulating Caryopsis Development of Rice (Oryza sativa L.)
Source: Plants (Basel). 2025 Jan 7;14(2):151. doi: 10.3390/plants14020151 (PMC11768212; doi:10.3390/plants14020151)
Supplement: Supplementary file 1 [file plants-14-00151-s001.zip › Gene family- Figures-5.pptx]

## Slide 1
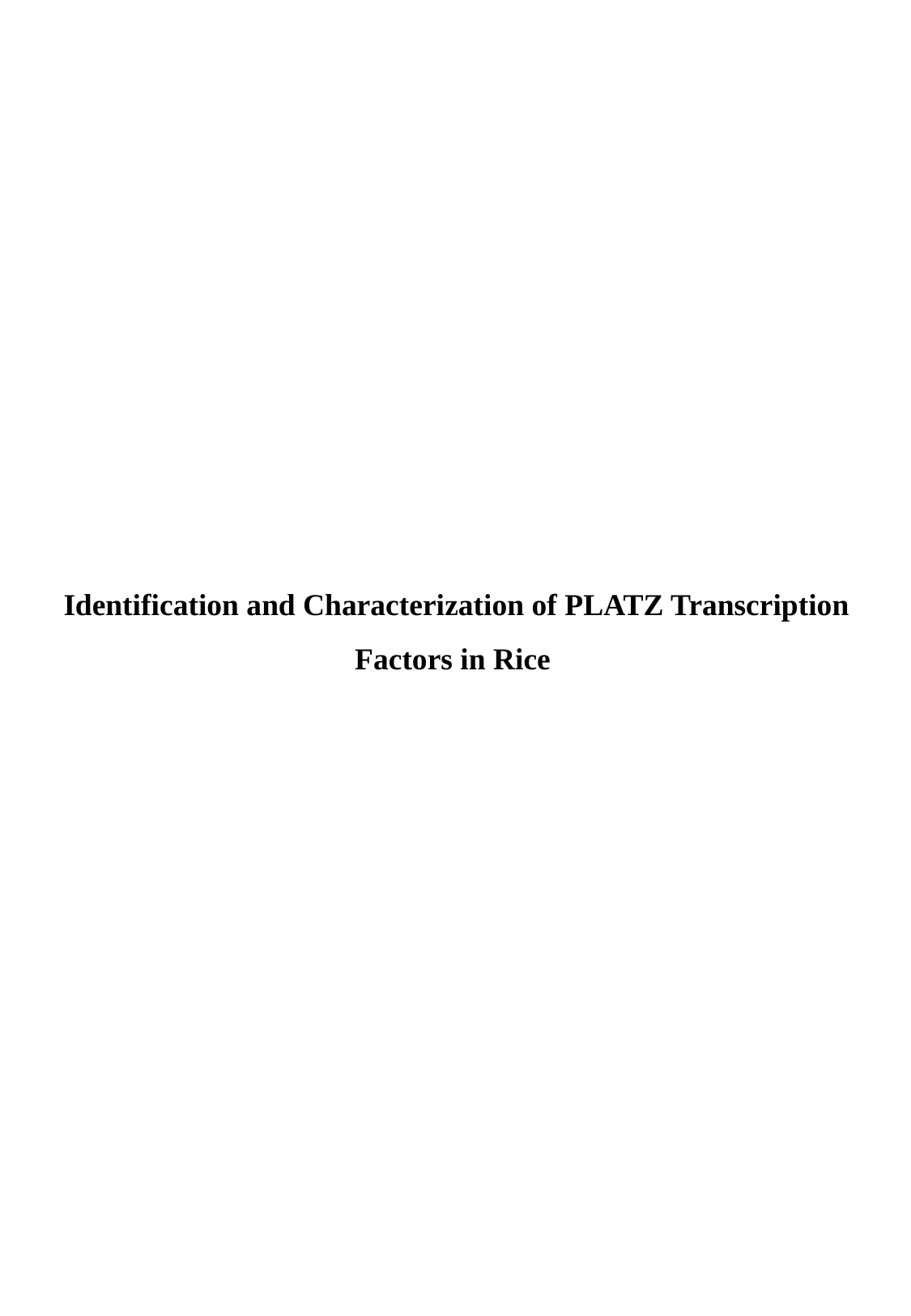

Identification and Characterization of PLATZ Transcription Factors in Rice

## Slide 2
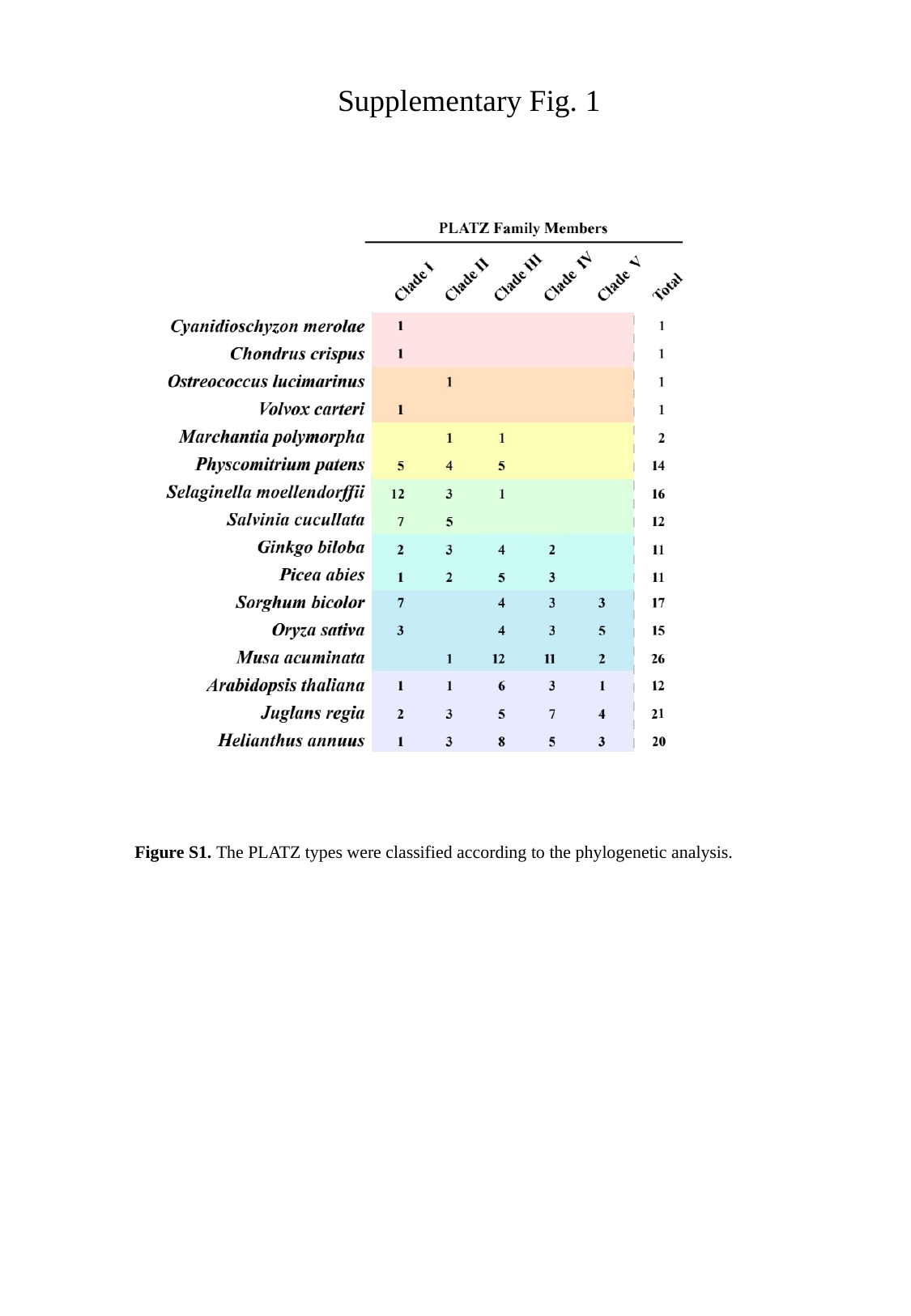

Supplementary Fig. 1
Figure S1. The PLATZ types were classified according to the phylogenetic analysis.

## Slide 3
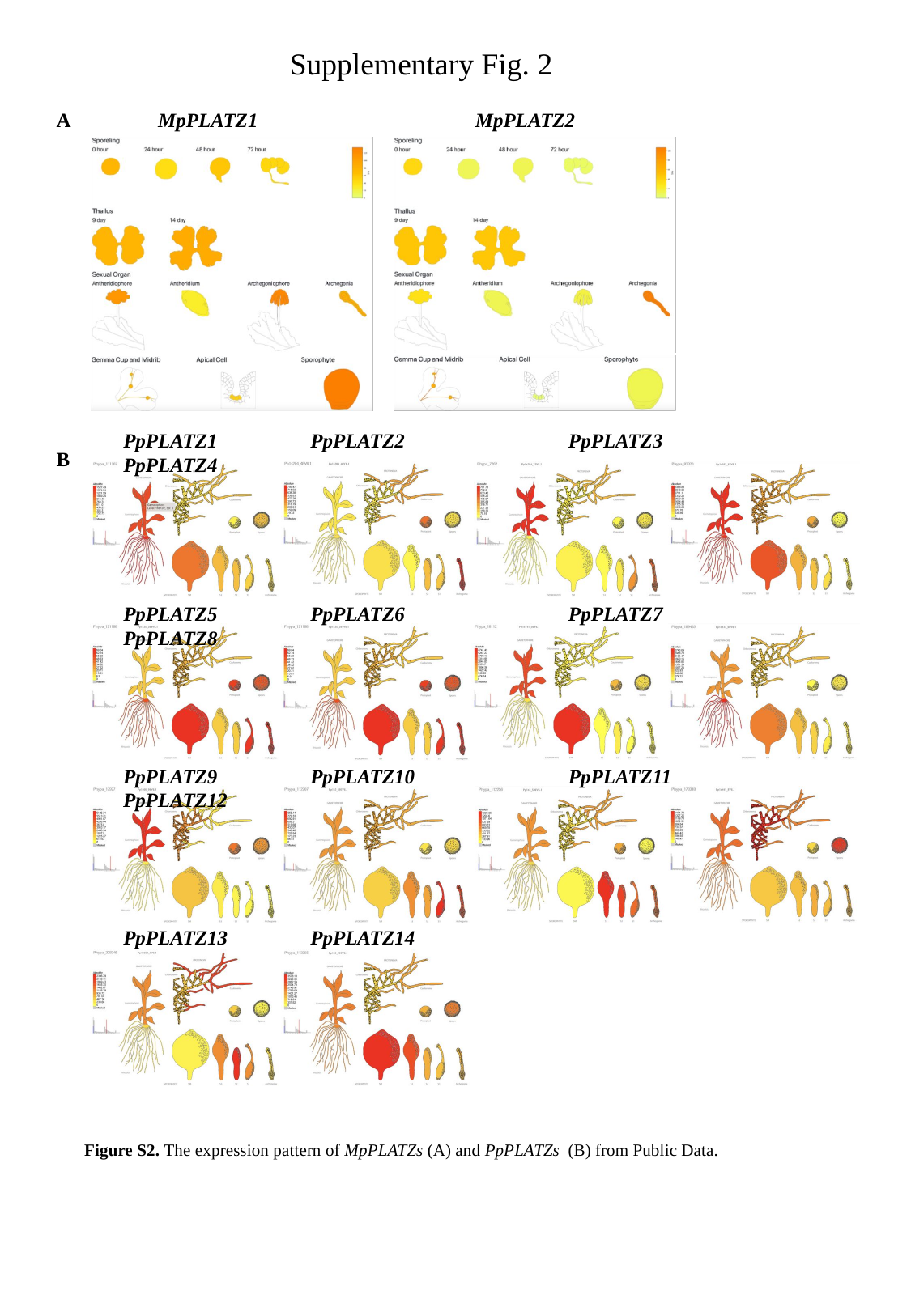

Supplementary Fig. 2
A
B
MpPLATZ1 MpPLATZ2
PpPLATZ1 	 PpPLATZ2 	 PpPLATZ3 	 PpPLATZ4
PpPLATZ5 	 PpPLATZ6 	 PpPLATZ7 	 PpPLATZ8
PpPLATZ9 	 PpPLATZ10 	 PpPLATZ11 PpPLATZ12
PpPLATZ13 	 PpPLATZ14
Figure S2. The expression pattern of MpPLATZs (A) and PpPLATZs (B) from Public Data.

## Slide 4
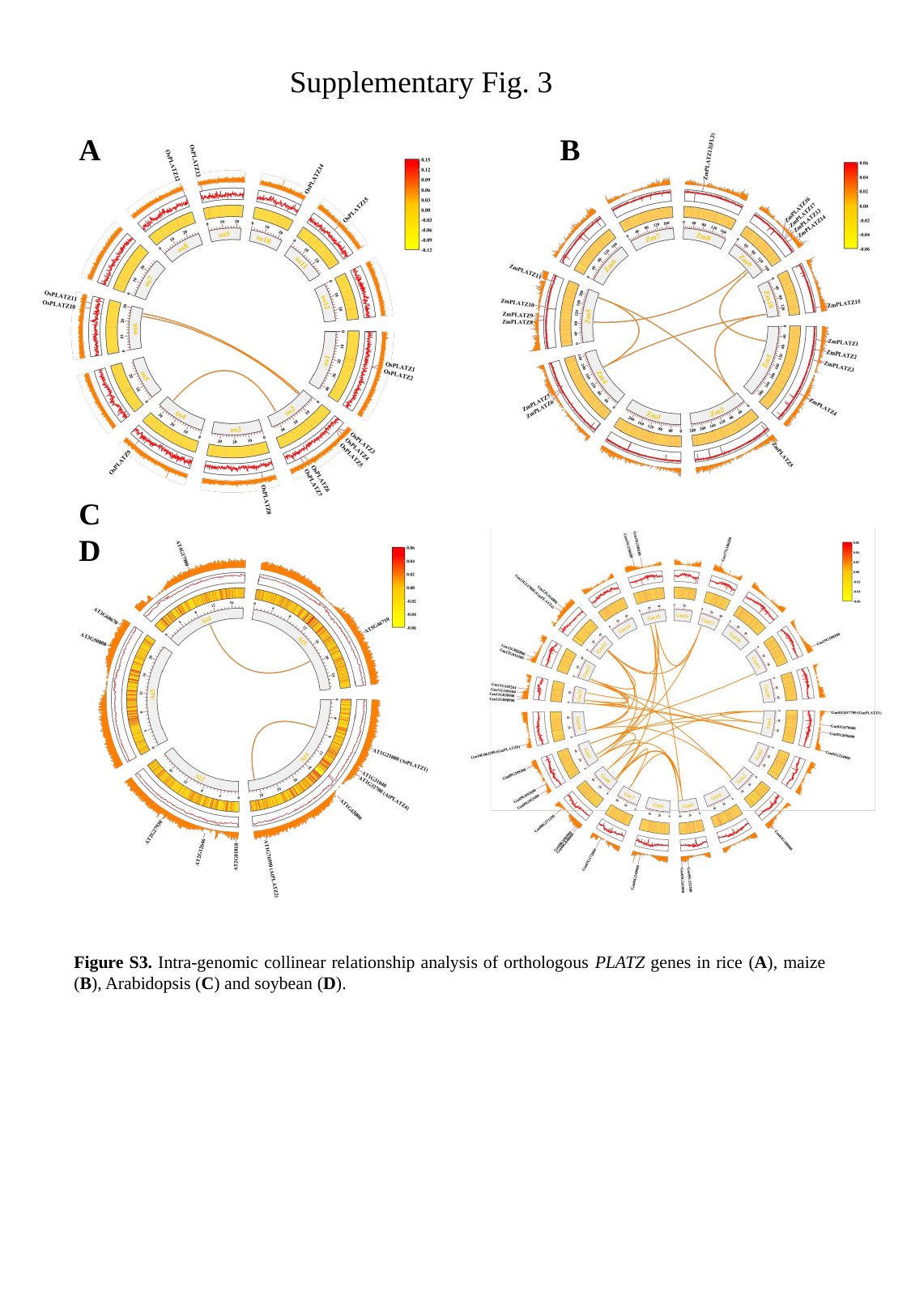

Supplementary Fig. 3
A B
C D
Figure S3. Intra-genomic collinear relationship analysis of orthologous PLATZ genes in rice (A), maize (B), Arabidopsis (C) and soybean (D).

## Slide 5
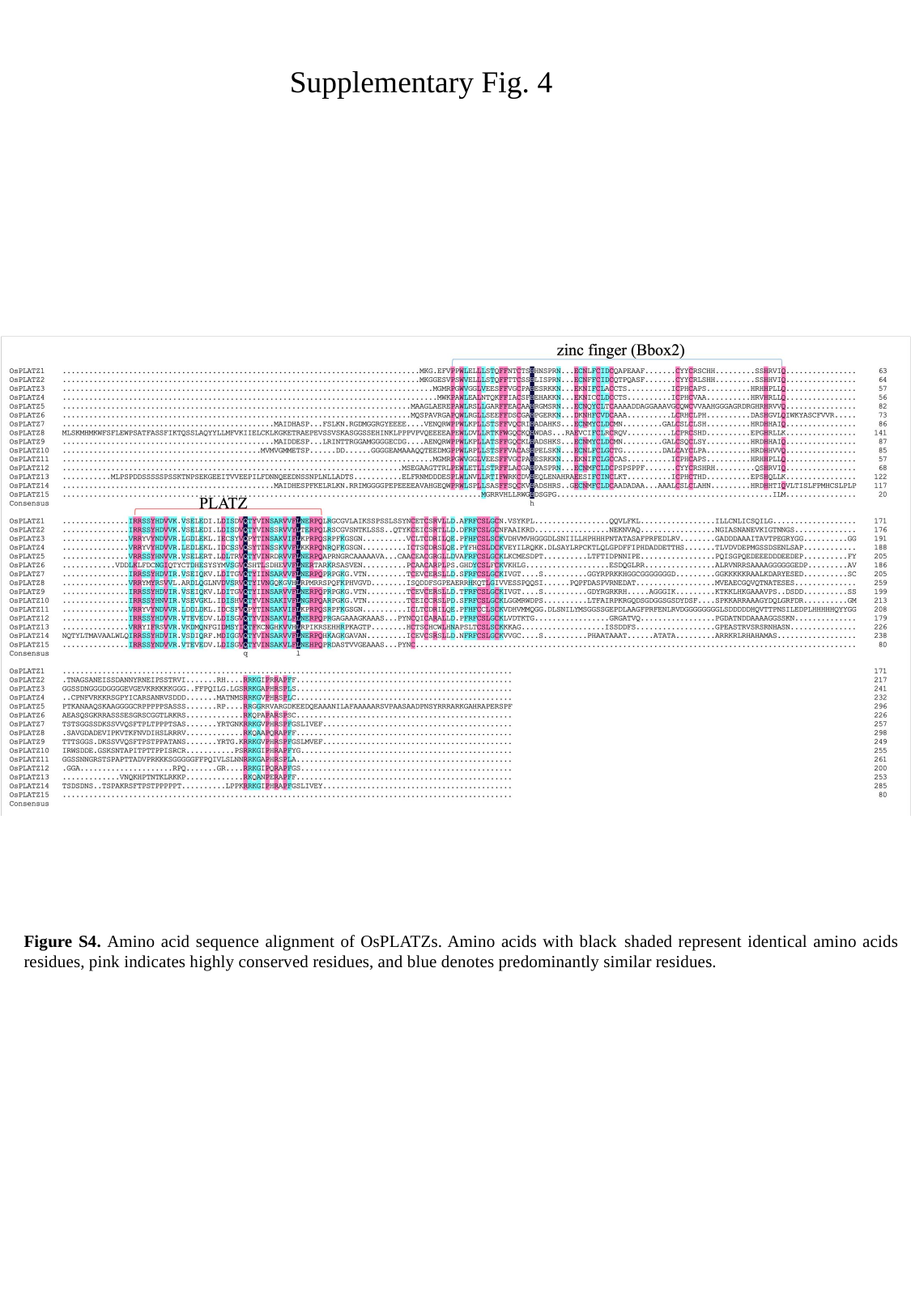

Supplementary Fig. 4
Figure S4. Amino acid sequence alignment of OsPLATZs. Amino acids with black shaded represent identical amino acids residues, pink indicates highly conserved residues, and blue denotes predominantly similar residues.

## Slide 6
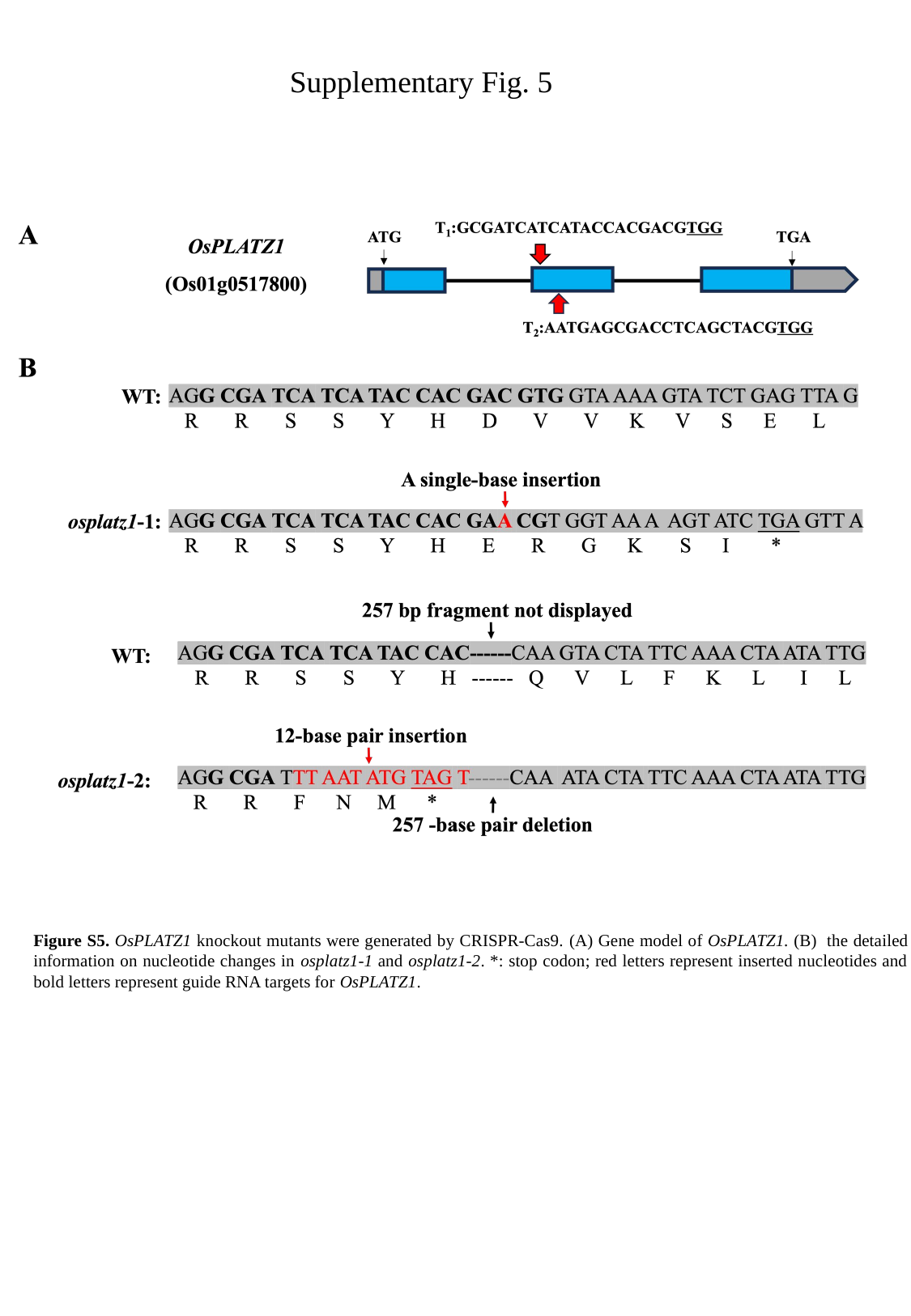

Supplementary Fig. 5
Figure S5. OsPLATZ1 knockout mutants were generated by CRISPR-Cas9. (A) Gene model of OsPLATZ1. (B) the detailed information on nucleotide changes in osplatz1-1 and osplatz1-2. *: stop codon; red letters represent inserted nucleotides and bold letters represent guide RNA targets for OsPLATZ1.
